# The Chinese Brief Cognitive Test: Normative Data Stratified by Gender, Age and Education

**DOI:** 10.3389/fpsyt.2022.933642

**Published:** 2022-07-04

**Authors:** Shuling Ye, Mengjuan Xie, Xin Yu, Renrong Wu, Dengtang Liu, Shaohua Hu, Yong Xu, Huanzhong Liu, Xijin Wang, Gang Zhu, Huaning Wang, Shaohong Zou, Tao Li, Wanjun Guo, Xiufeng Xu, Yuqi Cheng, Yi Li, Juan Yang, Min Peng, Nan Li, Chuan Shi

**Affiliations:** ^1^Peking University Institute of Mental Health (Sixth Hospital), Beijing, China; ^2^National Clinical Research Center for Mental Disorders (Peking University Sixth Hospital), Beijing, China; ^3^Key Laboratory of Mental Health, Ministry of Health (Peking University), Beijing, China; ^4^Institute of Mental Health of Second Xiangya Hospital, Central South University, Changsha, China; ^5^Division of Psychotic Disorders, Shanghai Mental Health Center, Shanghai Jiao Tong University School of Medicine, Shanghai, China; ^6^Department of Psychiatry, First Affiliated Hospital, Zhejiang University School of Medicine, Hangzhou, China; ^7^Department of Psychiatry, First Clinical Medical College/First Hospital of Shanxi Medical University, Taiyuan, China; ^8^Department of Psychiatry, Chaohu Hospital, Anhui Medical University, Hefei, China; ^9^The First Harbin Psychiatric Hospital, Harbin, China; ^10^Department of Psychiatry, The First Affiliated Hospital of China Medical University, Shenyang, China; ^11^Department of Psychiatry, Xijing Hospital, The Fourth Military Medical University, Xi'an, China; ^12^Department of Clinical Psychology, People's Hospital of Xinjiang Uygur Autonomous Region, Urumqi, China; ^13^Affiliated Mental Health Center and Hangzhou Seventh People's Hospital, Zhejiang University School of Medicine, Hangzhou, China; ^14^The Mental Health Center, West China Hospital, Sichuan University, Chengdu, China; ^15^Department of Psychiatry, First Affiliated Hospital of Kunming Medical University, Kunming, China; ^16^Wuhan Mental Health Center, Tongji Medical College of Huazhong University of Science and Technology, Wuhan, China; ^17^Department of Psychology, Hainan Medical University, Haikou, China; ^18^Huazhong University of Science and Technology Union Shenzhen Hospital, Shenzhen, China; ^19^Research Center of Clinical Epidemiology, Peking University Third Hospital, Beijing, China

**Keywords:** schizophrenia, cognition, C-BCT, Chinese, MCCB

## Abstract

The aim of this study was to develop a brief version of cognitive assessment test for evaluating the efficacy of treatments targeting cognitive impairments in Chinese schizophrenia patients, to examine its reliability, and establish normative data. Stratified according to age, gender, and educational level, healthy adult subjects were recruited from fifteen institutions in seven administrative regions of China and 723 valid samples were obtained, of which 50 were retested. Generalized Linear Models were conducted to analyze the effects of age, sex, and education. There was no significant difference between genders, while significant effects were demonstrated respectively among age and education on the normative data of C-BCT. The Cronbach α of C-BCT is 0.75, and the test-retest reliability (ICC) ranged from 0.62 to 0.76. Normative data of C-BCT were generated by gender, age and education, and the effects of these demographic factors were analyzed. It revealed good internal consistency and test-retest reliability of C-BCT.

## Background

Cognitive impairment is one of the core features of schizophrenia, and almost all patients show different degrees of cognitive impairments (1). Studies have shown that average impairment in multiple domains of cognition in schizophrenia can reach 1–2 standard deviations below healthy controls in learning and memory, attention, speed of information processing, executive function, and social cognition (1). Cognitive function is clearly correlated with social function, and neurocognition accounts for ~20–60% of the variables that influence functional outcomes in patients with schizophrenia (2). Cognitive impairment, which is an important cause of functional disability and an important factor predicting other outcomes, has a significant impact on patients' quality of life (3). As new treatments are being developed to improve cognition in schizophrenia, instruments for assessing cognition are of utmost importance (4).

A number of tools have been developed to evaluate cognitive function in schizophrenia, varying in length, operability, test-retest reliability, assessment area, and sensitivity to treatment. The MATRICS consensus cognitive test battery (MCCB), which is widely used in the clinical and research field, is considered the “gold standard” for investigating cognitive-enhancing medications for schizophrenia (5). It comprises ten renowned subtests that assess seven cognitive domains, including speed of processing, attention/vigilance, working memory, verbal learning, visual learning, problem-solving, and social cognition (6). According to a large-scale meta-analysis in China, patients with first-episode schizophrenia (FES) show neurocognitive deficits across all seven MCCB cognitive domains, particularly in the speed of processing and attention/ vigilance. Symbol Coding and Trail Making Test may be the most sensitive tests to detect cognitive deficit in Chinese patients with FES (7).

MCCB has been translated into Chinese, and co-norming and standardization have been done in China, revealing sufficient clinical validity and reliability in controls and patients with schizophrenia (8). Yet, usually more than 60 min are needed to complete the test, even for healthy participants. And it needs trained neuropsychological professionals to guide the assessment, which substantially limits the assessment of schizophrenia patients in routine clinical work. Hurford et al. (4) developed A Brief Cognitive Assessment Tool for Schizophrenia (B-CATS), which consists of 4 tests with a mean administration time of 10.3 min. The correlation between B-CATS and MCCB was reported to be 0.76, and there was no significant difference between the two tests in functional capacity (4). The introduction of B-CATS made it possible to rapidly evaluate the overall cognitive function of patients with schizophrenia. Still, as B-CATS was developed based on foreign population samples, it is not clear whether it can be used in the Chinese cultural environment and whether it is sensitive and effective to Chinese schizophrenia patients. Therefore, it is necessary to develop a set of brief cognitive assessment tools suitable for Chinese cultural characteristics and sensitive to the detection of cognitive function in Chinese schizophrenia patients.

A meta-analysis was conducted by searching and summarizing the literature on cognitive assessments of schizophrenia in both English and Chinese database, including Pubmed, Embase, PsycINFO, China National Knowledge Infrastructure (CNKI), WANFANG DATA, WEIPU Journal Net (VIP), and Sino Biomedicine Service System (SinoMed). In combination with our clinical research data, Delphi method was used to conduct two rounds of consultation on the ten candidate cognitive tests with 30 professional psychiatrists who had rich clinical experiences and were engaged in neurocognitive function research (9). Concerning discriminative sensitivity, intercultural efficacy, reusability, acceptability of subjects, and ease of digitization, four tests were finally selected, comprising TMT-A, Symbol Coding, Continuous Performance Test and Digit Span (9). These four tests were turned into an electronic version that could be operated on smartphones and tablets, thus making up the Chinese Brief Cognitive Test (C-BCT). Moreover, it was estimated that it takes about 15 min to finish the assessment, which means it provides a more available and applicable assessment tool for schizophrenia.

As is acknowledged by extensive studies, demographic variables have strong influences on cognitive function (10, 11). According to research at home and abroad, gender, age and education have significant effects on cognitive function (8, 12, 13). It shows no significant difference in the overall level of cognition between men and women, whereas there are still differences in various cognitive domains (8, 12, 13). For example, men have advantages on visual space, while women perform better on verbal tasks (10, 11, 14). Besides, the influence of age on cognition could not be underestimated. With the increase of age, the cognitive functions of memory, attention and language ability will decline, and aging is the biggest risk factor for Alzheimer's disease (15–17). However, education has some of the largest affect across cognitive tasks among the demographic variables (11). Several studies have shown that higher education is associated with better global cognitive function, but not all the domain-specific cognitive function such as the speed of information processing (11, 18–22). Therefore, in this study, we took gender, age and education as stratified factors to establish norms and analyzed their effects, respectively.

## Materials and Methods

### Participants

The sample was recruited across seven administrative regions and 15 institutions in China: North of China by IMHPU and First Hospital of Shanxi Medical University; East of China by Shanghai Mental Health Center, Chaohu Hospital of Anhui Medical University, and the First Affiliated Hospital, Zhejiang University School of Medicine; North East of China by the First Harbin Psychiatric Hospital and the First Hospital of China Medical University; North West of China by Xijing Hospital and People's Hospital of Xinjiang Uygur Autonomous Region; South West of China by West China Hospital of Sichuan University and the First Affiliated Hospital of Kunming Medical University; Central China by Wuhan Mental Health Center and the Second Xiangya Hospital of Central South University; South of China by Hainan Medical University and Huazhong University of Science and Technology Union Shenzhen Hospital. The sample was stratified by age, gender, and education as reported by China Census Bureau in 2010 (23). A total of 759 healthy adult volunteers were assessed with both MCCB and C-BCT, and finally 723 valid samples were obtained. Four age groups: 18–29, 30–39, 40–49, 50–59, were considered by the corresponding proportions of subjects according to the age distribution of the Chinese population. Following the length of education, the subjects were divided into three groups: below 10 years (junior high school level or below), 10–12 years (senior high school level), and over 12 years (university level or above). After 2–4 weeks, around 8% of the subjects were randomly selected and retested by C-BCT, finally generating 50 valid samples. The participants received 100 RMB for transportation each time they attended the study.

### Recruitment Procedures

The recruitment procedure was identical in all 15 institutions. In each institution, the potential subjects were informed of the study through referrals and information sheets posted in the community. Participants were included if they were aged 18–59 years; were from one of seven administrative regions of China; if they were capable of reading and understanding Mandarin, and if they had at least 5 years of education.

Participants were excluded if they had a diagnosis of schizophrenia, bipolar disorder, depressive disorder, obsessive-compulsive disorder, post-traumatic stress disorder, attention deficit hyperactivity disorder, or other psychotic disorder; if they had a clinically significant neurological disease or head injury with loss of consciousness over an hour; if they had a diagnosis of mental retardation or pervasive developmental disorder; if they were currently taking antipsychotics, antidepressants, anti-anxiety drugs, cognition-enhancing medication or used anesthetics for pain within the last 72 h; if they had a history of alcohol or drug abuse over 10 years; if they had any substance use in the last 3 days prior to testing; if they had more than four alcoholic drinks per day for each of the last 3 days prior to testing; if they were not able to understand Mandarin sufficiently to comprehend testing instructions; or if they could not appropriately comprehend the consent form.

The details of the study were explained to the eligible participants. All subjects who agreed to participate signed the informed consents, and they were put into the respective subgroups according to their age, gender, and education level until the recruitment cells were filled. All procedures were approved by the IRB of IMHPU.

### Study Design

The study involved 15 institutions. To ensure appropriate and consistent practice, relevant standardized trainings of operation procedure were arranged at the beginning of the study. Trained neuropsychological examiners in each institution conducted a structured interview to acquire the subjects' demographic data and guided the C-BCT tests in following order:

(1) Trail Making Test, Part A (TMT-A): the respondent is required to connect consecutive numbers that are arranged in irregular locations on the electronic screen. If the respondent makes a mistake, the system will automatically recognize, remind him/her and then continue. The score represents the time taken to complete the task. This test mainly measures the cognitive areas involving the speed of information processing, visual scanning, and cognitive flexibility.

(2) Symbol Coding: in this test, the oracle bone scripts are used as symbols, which also symbolized Chinese cultural characteristics. The respondent is required to pair the numbers with corresponding oracle symbols as quickly as possible for a 90-s period. The number of correct answers will be counted as the total score. This test mainly measures attention, the speed of information processing, and the executive function of transformation.

(3) Continuous Performance Test (CPT): in this test, the Chinese zodiac was used as stimulus-responsive materials, as it is more suitable for the Chinese cultural environment. A total of three groups of different animal combinations are briefly and continuously played on the electronic device. The respondent is required to click each time that two stimuli in a row are identical. D-prime is used as the main evaluation index, which is a standard score that reflects the ability of subjects to distinguish between targets and distractions. This test mainly measures sustained and focused attention.

(4) Digit Span: this test adopts the form of voice broadcast with keystroke answers, which makes the operation simple and feasible. Ranging from two to nine digits, sequences of increasing length are played at a certain speed. For Digit Span Forward, the respondent is required to type the sequence of numbers in the same order as they are just played. For Digit Span Backward, the respondent is required to type the sequence of numbers in reverse order. This test mainly reflects the ability of auditory verbal working memory.

In addition, a minimum level of psychometric administration guidance was required when preparing the cognitive tests. For example, the participants were informed to respond appropriately (no rushing through by randomly responding), and independently from others. The purpose of the cognitive battery was explained in advance and the participants should put forth sufficient effort.

### Statistical Analysis

All recruited participants were assessed with both MCCB and C-BCT. We just analyzed the normative data of C-BCT in this article. The raw scores of the four subtests were standardized to scaled scores based on the 723 subjects so that each test had a mean of 10 and a standard deviation of 3 in the sample. The composite scaled score was generated from taking the mean of four subtests scaled scores and then re-standardized so that it had a mean of 0 and a standard deviation of 1. Data analyses were performed using the IBM SPSS Statistics 22 program.

The scaled scores for the four subtests and the composite scaled score were analyzed to assessed age, gender and education effects. Take into account the possible interaction or confounding effects among age, sex and years of education, we conducted Generalized Linear Models (GLMs) to analyze the differences in performance related to age, gender, and education. Cronbach's alpha was used to evaluate internal reliability for the overall test battery and Intraclass Correlation Coefficient (ICC) for test-retest reliability of each subtest.

## Results

### Demographic Characteristics

The Demographic characteristics of the normative sample were shown on (Table 1). A total of 723 valid samples of healthy adult subjects were enrolled in the study, with an average age of 37.0 years. Among the subjects, there were 374 males and 349 females (gender ratio of 1.07:1). The highest education level was 20 years, and the lowest was 5 years, with an average education level of 10 years which is in secondary middle school. Ten subjects were left-handed, and the remaining 713 were right-handed. Among the subjects, 69.3% of them were married, 28.2% were unmarried, 2.2% were divorced, and 0.3% were widowed. As for the employment status, 81.1% of the subjects were employed, 16.9% were unemployed, 1.5% were retired, and 0.5% lost their jobs.

**Table 1:** Demographic characteristics of the normative sample.

	**All subjects**	***N*** **= 723**
Age	37.02	
Education	10.28	
Gender	Male	374 (51.7%)
	Female	349 (48.3%)
Marriage	Unmarried	204 (28.2%)
	Married	501 (69.3%)
	Divorced	16 (2.2%)
	Widowed	2 (0.3%)
Dominant hand	Left-handed	10 (1.4%)
	Right-handed	713 (98.6%)
Employment	Employed	586 (81.1%)
	Unemployed	122 (16.9%)
	Out of work	4 (0.5%)
	Retired	11 (1.5%)

As was shown in the analysis results of the GLMs, there was no interaction among age, gender and years of education on the four subtests as well as the composite scaled score in our normative data.

### Gender Effects

As was revealed by Generalized Linear Models, there was no statistically significant difference between genders on the four subtests as well as the composite scaled score (*p* > 0.05) (Table 2).

**Table 2:** Comparison between genders on the four subtests and the composite scaled score.

	**Gender**	**Mean**	**SD**	**B**	**Std. Error**	***P***	**95%Wald CI**	
							**Lower**	**Upper**
TMT-A	Female	9.74	3.29	0.45	0.75	0.549	−1.924	1.023
	Male	10.25	2.69					
Symbol coding	Female	9.83	2.95	0.06	0.72	0.939	−1.465	1.354
	Male	10.16	3.04					
CPT	Female	9.86	2.89	−0.48	0.78	0.534	−1.042	2.011
	Male	10.13	3.09					
Digit Span	Female	9.94	3.28	0.08	0.77	0.923	−1.581	1.432
	Male	10.06	2.71					
Composite scaled score	Female	−0.05	0.80	0.01	0.18	0.964	−0.352	0.336
	Male	0.05	0.71					

### Age Effects

Generalized Linear Models showed significant age effects for the four subtests as well as the composite scaled score (Table 3). Generally speaking, the youngest group, the 18–29-year-old group, presented the best performance on all four subtests as well as the composite scaled score, and the 30–39-year-old group ranked in second place, with no significant difference between groups aged 40–49 and 50–59 on the four subtests as well as the composite scaled score. Additionally, in the TMT-A and CPT subtests, there was no statistically significant difference between 18–29 and 30–39 group.

Table 3**(A)** Comparison among education groups on the four subtests and the composite scaled score; **(B)** *p*-values of pairwise comparisons among education groups from Generalized Linear Models.

**Table:** 

**(A)**							
	**Years of education**	**Mean**	**SD**	**B**	**Std. Error**	**95%Wald CI**	
						**Lower**	**Upper**
TMT-A	<10	9.39	3.38	−1.689	1.0096	−3.667	0.290
	10–12	10.60	2.11	−0.199	1.1281	−2.410	2.012
	>12	11.51	1.42	0			
Symbol Coding	<10	9.40	2.93	−3.224	0.9657	−5.117	−1.331
	10–12	10.19	2.54	−2.182	1.0790	−4.297	−0.067
	>12	12.19	2.96	0			
CPT	<10	9.44	3.15	−0.921	1.0457	−2.970	1.129
	10–12	10.50	2.74	−0.303	1.1684	−2.593	1.987
	>12	11.49	1.93	0			
Digit Span	<10	9.29	2.95	−1.489	1.0322	−3.512	0.534
	10–12	10.54	2.67	−0.983	1.1533	−3.243	1.278
	>12	12.06	2.56	0			
Composite scaled score	<10	−0.21	0.77	−0.610	0.2359	−1.073	−0.148
	10–12	0.15	0.60	−0.306	0.2636	−0.822	0.211
	>12	0.60	0.51	0

**Table:** 

**(B)**					
	**TMT-A**	**Symbol coding**	**CPT**	**Digit span**	**Composite scaled score**
Aged 18–29 and 30–39	0.151	0.043	0.291	0.015	0.013
Aged 18–29 and 40–49	<0.001	<0.001	<0.001	<0.001	<0.001
Aged 18–29 and 50–59	<0.001	<0.001	<0.001	<0.001	<0.001
Aged 30–39 and 40–49	0.001	<0.001	0.010	0.039	<0.001
Aged 30–39 and 50–59	<0.001	<0.001	<0.001	0.013	<0.001
Aged 40–49 and 50–59	0.158	0.914	0.202	0.554	0.220

### Education Effects

Statistical results revealed that education had a significant effect on all four subtests and the composite scaled score (*p* < 0.05, Table 4). Generally speaking, a higher educational level was associated with improved cognitive function in different domains, as well as with general cognitive function. However, there was no statistically significant difference in TMT-A between moderate education (10–12 years) and higher education (>12 years), nor in Symbol Coding between lower education (<10 years) and moderate education (10–12 years).

Table 4**(A)** Comparison among age groups on the four subtests and the composite scaled score; **(B)** *p*-values of pairwise comparisons among age groups from Generalized Linear Models.

**Table:** 

**(A)**							
	**Age group (years)**	**Mean**	**SD**	**B**	**Std. Error**	**95%Wald CI**	
						**Lower**	**Upper**
TMT-A	18–29	11.32	1.59	2.521	1.0219	0.518	4.524
	30–39	10.47	2.29	2.417	1.1458	0.171	4.663
	40–49	8.88	3.55	1.017	1.2791	−1.490	3.524
	50–59	8.30	3.72	0			
Symbol Coding	18–29	11.56	2.55	1.544	0.9775	−0.372	3.460
	30–39	10.48	2.93	1.070	1.0960	−1.078	3.218
	40–49	8.69	2.38	−1.196	1.2235	−3.594	1.202
	50–59	8.07	2.86	0			
CPT	18–29	11.15	2.28	1.912	1.0585	−0.163	3.986
	30–39	10.24	2.62	2.476	1.1868	0.150	4.802
	40–49	9.06	3.37	0.840	1.3249	−1.757	3.437
	50–59	8.71	3.29	0			
Digit Span	18–29	11.22	2.87	2.364	1.0448	0.316	4.412
	30–39	9.89	2.92	1.512	1.1715	−0.784	3.808
	40–49	9.13	2.77	−0.333	1.3078	−2.896	2.230
	50–59	8.93	2.84	0			
Composite scaled score	18–29	0.44	0.52	0.695	0.2388	0.227	1.163
	30–39	0.09	0.65	0.623	0.2677	0.098	1.148
	40–49	−0.35	0.75	0.027	0.2989	−0.558	0.613
	50–59	−0.50	0.78	0

**Table:** 

**(B)**					
**TMT-A**	**Symbol coding**	**CPT**	**Digit span**	**Composite scaled score**	
<10 and 10–12 years	0.003	0.283	0.022	0.001	0.001
<10 and >12 years	<0.001	<0.001	<0.001	<0.001	<0.001
10–12 and >12 years	0.115	<0.001	0.016	0.009	<0.001

### Reliability

It revealed a good internal consistency of C-BCT (Cronbach's alpha = 0.75), which reflected considerable coherence of the components of C-BCT. To measure test-retest reliability, around eight percent of the subjects were randomly selected and required to be retested in 2–4 weeks. Finally 50 valid samples were obtained. The Intraclass Correlation Coefficient (ICC) was conducted as a test-retest reliability estimate of four subtests as well as the composite scaled score. We observed the highest ICC on the Composite scaled score (0.76), followed by Digit Span (0.73), Symbol Coding (0.71), CPT (0.67), and the lowest on TMT-A (0.62).

## Discussion

The Chinese Brief Cognitive Test (C-BCT) was developed as a more available and applicable assessment tool for schizophrenia. In this study, we collected and analyzed the normative data in China. In the analysis of demographic effects, we found no interaction among age, gender and years of education in our normative data. Our results showed a significant effect among age and education, which is consistent with the standardized English version of MCCB in the US and the Chinese version in China (8, 12). However, there was no significant difference between genders. Besides, we demonstrated good reliability in subtests and the overall test battery.

In terms of gender effect, our study showed no statistically significant difference between men and women on TMT-A, CPT and the Composite scaled score, which was consistent with the standardized English version of MCCB in the US and the Chinese version in China (8, 12). Women tended to outperform men on both Symbol Coding and Digit Span, whereas the difference was not statistically significant. This result was inconsistent with the original MCCB in the US or the MCCB in China, and we considered that the design and compilation of these two subtests in the C-BCT were different from the original version. Firstly, as for Symbol Coding in the C-BCT, Oracle is used as the symbol information, with higher requirements for visual learning and speed of information processing. In the Chinese version of MCCB in China, women significantly performed better than men on Symbol Coding, whereas men had better visual learning ability (8). Secondly, unlike Spatial Span in the MCCB, Digit Span in the C-BCT measures not only auditory working memory, but it also involves verbal ability. Even if men performed better on Spatial Span in the original version of MCCB as well as the Chinese version, meanwhile it revealed that women had better verbal ability (8, 12). Hence we considered these two points might attribute to the inconsistency.

As is well-known, age has an important effect on cognitive test scores, and the normal aging process is associated with declines in certain cognitive abilities, such as the speed of information processing, some certain memory, language, visual space, and executive functioning (10, 11, 15, 24). Regarding the age effect on the C-BCT, the performance in most cognitive domains descended with the increasing age in general. However, there was no significant difference between some age groups in certain cognitive domains, which was observed in the original version of MCCB, the Chinese version of MCCB and the C-BCT (8, 12). In the normative data of the C-BCT, there was no significant difference between groups aged 18–29 and 30–39 on TMT-A and CPT. TMT-A mainly measures speed of information processing, visual scanning and cognitive flexibility. CPT mainly reflects attention/vigilance. This result revealed that these two age groups of healthy subjects did not significantly differ in the cognitive domains involved. Besides, it revealed no statistically difference between group aged 40–49 and 50–59 on the four subtests as well as the Composite scaled score, which suggested that it's relatively stable from the age of 40–59 in the cognitive function covered by the C-BCT.

With respect to the education effect, a higher level of education was undoubtedly associated with better cognitive performance (11). Although there was a gradual upward trend on the four subtests and Composite scaled score with the years of education increasing, we found no statistically significant difference between the moderate education level (10–12 years) and the higher education level (>12 years) in TMT-A, nor between the lower education level (<10 years) and the moderate education level (10–12 years of education) in Symbol Coding. The core cognitive domain of both TMT-A and Symbol Coding is the speed of information processing. It's not only related to age and education level but also the innate neuro-reactivity, which cannot be further improved after being raised to a certain extent by education. Therefore, the results of TMT-A suggests that the competence of speed of information processing has reached a relatively constant level with high school education, and continued academic training cannot lead to further significant improvement. In terms of Symbol Coding, it requires the ability of working memory on the basis of speed of information processing. The results showed no significant difference between primary school education and middle school education, whereas academic training at the university seems very helpful in this area.

Similarly, as a brief cognition battery, the B-CATS was composed of Trail-Making Test A and B, Digit Symbol Substitution Test, and Animal Fluency (4). With an approximate administration time of 10 min, both B-CATS and C-BCT demonstrated good reliability. While both these two brief assessment tools can be administered by non-psychometrically trained clinicians, C-BCT is a digital tool, which can automatically collect, analyze and store data, and is also convenient for quality control. Besides, there is a lack of normative data for the complete B-CATS battery, including the lack of Chinese normative data (4). Moreover, the intercultural adaptability of each subtest of B-CATS in China is uncertain, as well as the sensitivity and effectiveness in Chinese schizophrenia patients. Considering the above-listed factors, we believe C-BCT is more applicable in China.

Another widely used cognitive tool for schizophrenia is the Brief Assessment of Cognition in Schizophrenia (BACS), with <35 min to administrate (25). A tablet-based version of the BACS called the BAC App has been developed, which generates consistent results (26). Moreover, Keefe et al developed a modified version for patients with affective disorders: the Brief Assessment of Cognition in Affective Disorder (BAC-A) (27). Wang et al developed the normative data of the Chinese version of both the BACS and the BAC-A among the Mandarin-speaking population in Taiwan, which lacked reliability and validity verified (28, 29). Hence, comparing to the BACS related instruments, the C-BCT is a digital, applicable and effective cognitive battery with Chinese norm established and reliability verified, which can quickly assess the global cognitive function and is appropriate for use in clinical trials as well as clinical practice.

There are several potential limitations in the present study. Firstly, the CPT in C-BCT needs to be improved, which reduce the operation time with relatively less number of tasks. With the stimulus material changed into animals, the subjects' familiarity with the Chinese zodiac is also uneven. Secondly, the results show no statistically difference between group aged 40–49 and 50–59 in the cognitive domains C-BCT covered, which probably suggests further optimization for the age stratification of the norm. The other limitation is the lack of assessment for other cognitive domains, such as social cognition which is viewed as an important domain of cognitive deficit as well as a good predictor of functional outcomes in schizophrenia. However, considering the primary purpose of this assessment tool, convenience and efficiency are crucial for clinical utility. Thus, detailed cognitive evaluation could be further improved.

In conclusion, C-BCT was developed as a brief Chinese version of MCCB for assessing cognitive improvement in schizophrenia. We established Chinese normative data stratified by gender, age and education, analyzed effects on these demographic factors, and demonstrated good reliability in subtests and the overall test battery, thus providing a mean for clinicians to briefly measure the global cognition during routine visits and accordingly making adjustments to treatment. We established the regression equation by converting it to the standardized T score. Criterion validity and empirical validity will be published in future studies, thus providing sufficient reference for clinical usage.

All subjects have also completed MCCB battery and the results will be reported in the future. Additionally, the C-BCT has currently been studied only in patients with schizophrenia, but the multiple cognitive function domains involved in C-BCT may also be applicable to evaluate the cognitive status of other mental disorders, which can be further explored in future studies.

## Data Availability Statement

The original contributions presented in the study are included in the article/Supplementary Material, further inquiries can be directed to the corresponding author/s.

## Ethics Statement

The studies involving human participants were reviewed and approved by Peking University Sixth Hospital. The patients/participants provided their written informed consent to participate in this study.

## Author Contributions

CS and YX designed the study, wrote the protocol, and managed the literature searches as well as analyses. SY, MX, RW, DL, SH, YX, HL, XW, GZ, HW, SZ, TL, WG, XX, YC, YL, JY, and MP selected the sample and evaluated patients. SY and CS contributed in the interpretation of results and wrote the first draft of the manuscript. SY and NL undertook the statistical analysis. All authors contributed to the article and approved the submitted version.

## Funding

This research was supported, respectively, in part by National Natural Science Foundation of China (Project Number: 82071509), National Natural Science Foundation of China (Project Number: 72110107003), Chinese Neuropsychological Normative Project (CN-NORM) among the middle-aged and elderly (Project Number: 2018YFC1314202), Jiangsu Nhwa Pharmaceutical Co., Ltd and National Natural Science Foundation of China (Project Number: 81701067).

## Conflict of Interest

The authors declare that the research was conducted in the absence of any commercial or financial relationships that could be construed as a potential conflict of interest. The handling editor TZ declared a shared parent affiliation with the author DL at the time of review.

## Publisher's Note

All claims expressed in this article are solely those of the authors and do not necessarily represent those of their affiliated organizations, or those of the publisher, the editors and the reviewers. Any product that may be evaluated in this article, or claim that may be made by its manufacturer, is not guaranteed or endorsed by the publisher.

## References

[1] McCleery A Ventura J Kern RS Subotnik KL Gretchen-Doorly D Green MF. Cognitive functioning in first-episode schizophrenia: MATRICS Consensus Cognitive Battery (MCCB) Profile of Impairment. Schizophr Res. (2014) 157:33–9. 10.1016/j.schres.2014.04.039 24888526 PMC4112962

[2] Green MF Kern RS Braff DL Mintz J. Neurocognitive deficits and functional outcome in schizophrenia: are we measuring the right stuff? Schizophr Bull. (2000) 26:119–36. 10.1093/oxfordjournals.schbul.a033430 10755673

[3] Green MF. What are the functional consequences of neurocognitive deficits in schizophrenia? Am J Psychiatry. (1996) 153:321–30. 10.1176/ajp.153.3.321 8610818

[4] Hurford IM Ventura J Marder SR Reise SP Bilder RM. A 10-minute measure of global cognition: Validation of the Brief Cognitive Assessment Tool for Schizophrenia (B-CATS). Schizophr Res. (2018) 195:327–33. 10.1016/j.schres.2017.08.033 28918221

[5] Green MF Nuechterlein KH Gold JM Barch DM Cohen J Essock S. Approaching a consensus cognitive battery for clinical trials in schizophrenia: the NIMH-MATRICS conference to select cognitive domains and test criteria. Biol Psychiatry. (2004) 56:301–7. 10.1016/j.biopsych.2004.06.023 15336511

[6] Nuechterlein KH Barch DM Gold JM Goldberg TE Green MF Heaton RK. Identification of separable cognitive factors in schizophrenia. Schizophr Res. (2004) 72:29–39. 10.1016/j.schres.2004.09.007 15531405

[7] Zhang H Wang Y Hu Y Zhu Y Zhang T Wang J. Meta-analysis of cognitive function in Chinese first-episode schizophrenia: MATRICS Consensus Cognitive Battery (MCCB) profile of impairment. Gen Psychiatry. (2019) 32:e100043. 10.1136/gpsych-2018-100043 31423473 PMC6677937

[8] Shi C Kang L Yao S Ma Y Li T Liang Y. The MATRICS Consensus Cognitive Battery (MCCB): co-norming and standardization in China. Schizophr Res. (2015) 169:109–15. 10.1016/j.schres.2015.09.003 26441005 PMC4916953

[9] Ma K Yu X Li C-B Shi C. A Delphi study of the brief cognitive assessment for schizophrenia. Chin Ment Health J. (2020) 34:736–40. 10.3969/j.issn.1000-6729.2020.9.003

[10] Wiederholt WC Cahn D Butters NM Salmon DP Kritz-Silverstein D Barrett-Connor E. Effects of age, gender and education on selected neuropsychological tests in an elderly community cohort. J Am Geriatr Soc. (1993) 41:639–47. 10.1111/j.1532-5415.1993.tb06738.x 8505462

[11] Rexroth DF Tennstedt SL Jones RN Guey LT Rebok GW Marsiske MM. Relationship of demographic and health factors to cognition in older adults in the ACTIVE study. J Aging Health. (2013) 25:128S−46. 10.1177/0898264313498415 24385633 PMC4116102

[12] Kern RS Nuechterlein KH Green MF Baade LE Fenton WS Gold JM. The MATRICS Consensus Cognitive Battery, part 2: co-norming and standardization. Am J Psychiatry. (2008) 165:214–20. 10.1176/appi.ajp.2007.07010043 18172018

[13] Rodriguez-Jimenez R Bagney A Garcia-Navarro C Aparicio AI Lopez-Anton R Moreno-Ortega M. The MATRICS consensus cognitive battery (MCCB): co-norming and standardization in Spain. Schizophr Res. (2012) 134:279–84. 10.1016/j.schres.2011.11.026 22192501

[14] Van Der Elst W Van Boxtel MPJ Van Breukelen GJP Jolles J. Rey's verbal learning test: normative data for 1855 healthy participants aged 24–81 years and the influence of age, sex, education, and mode of presentation. J Int Neuropsychol Soc. (2005) 11:290–302. 10.1017/S1355617705050344 15892905

[15] Harada CN Natelson Love MC Triebel KL. Normal cognitive aging. Clin Geriatr Med. (2013) 29:737–52. 10.1016/j.cger.2013.07.002 24094294 PMC4015335

[16] Rönnlund M Nyberg L Bäckman L Nilsson L-G. Stability, growth, and decline in adult life span development of declarative memory: cross-sectional and longitudinal data from a population-based study. Psychol Aging. (2005) 20:3–18. 10.1037/0882-7974.20.1.3 15769210

[17] Keller JN. Age-related neuropathology, cognitive decline, and Alzheimer's disease. Ageing Res Rev. (2006) 5:1–13. 10.1016/j.arr.2005.06.002 16084778

[18] Zahodne LB Nowinski CJ Gershon RC Manly JJ. Which psychosocial factors best predict cognitive performance in older adults? J Int Neuropsychol Soc. (2014) 20:487–95. 10.1017/S1355617714000186 24685143 PMC4493753

[19] Jefferson AL Gibbons LE Rentz DM Carvalho JO Manly J Bennett DA. Life course model of cognitive activities, socioeconomic status, education, reading ability, and cognition. J Am Geriatr Soc. (2011) 59:1403–11. 10.1111/j.1532-5415.2011.03499.x 21797830 PMC3222272

[20] Lavrencic LM Churches OF Keage HAD. Cognitive reserve is not associated with improved performance in all cognitive domains. Appl Neuropsychol Adult. (2018) 25:473–85. 10.1080/23279095.2017.1329146 28594578

[21] Ritchie SJ Bates TC Der G Starr JM Deary IJ. Education is associated with higher later life IQ scores, but not with faster cognitive processing speed. Psychol Aging. (2013) 28:515–21. 10.1037/a0030820 23276218

[22] Li X Song R Qi X Xu H Yang W Kivipelto M. Influence of cognitive reserve on cognitive trajectories: role of brain pathologies. Neurology. (2021) 97:e1695–706. 10.1212/WNL.0000000000012728 34493618 PMC8605617

[23] China Census Bureau C. China Population in 2010. Beijing (2011).

[24] Salthouse TA. What and when of cognitive aging. Curr Dir Psychol Sci. (2004) 13:140–4. 10.1111/j.0963-7214.2004.00293.x

[25] Keefe RSE Goldberg TE Harvey PD Gold JM Poe MP Coughenour L. The Brief Assessment of Cognition in Schizophrenia: reliability, sensitivity, and comparison with a standard neurocognitive battery. Schizophr Res. (2004) 68:283–97. 10.1016/j.schres.2003.09.011 15099610

[26] Atkins AS Tseng T Vaughan A Twamley EW Harvey P Patterson T. Validation of the tablet-administered Brief Assessment of Cognition (BAC App). Schizophr Res. (2017) 181:100–6. 10.1016/j.schres.2016.10.010 27771201

[27] Keefe RSE Fox KH Davis VG Kennel C Walker TM Burdick KE. The Brief Assessment of Cognition In Affective Disorders (BAC-A): performance of patients with bipolar depression and healthy controls. J Affect Disord. (2014) 166:86–92. 10.1016/j.jad.2014.05.002 25012414

[28] Lee C-Y Lee S-Y Huang Y-C Hung C-F Lee Y Lee M-I. The Chinese version of the Brief Assessment of Cognition in Affective Disorders: normative data of a Mandarin-speaking population. Clin Neuropsychol. (2018) 32:1–14. 10.1080/13854046.2017.1400108 29108470

[29] Wang L-J Huang Y-C Hung C-F Chen C-K Chen Y-C Lee P-Y The Chinese version of the brief assessment of cognition in schizophrenia: data of a large-scale Mandarin-speaking population. Arch Clin Neuropsychol. (2017) 32:289–96. 10.1093/arclin/acw100 28431029

